# Criteria for Referring Patients With Outpatient Gastroenterological Disease for Specialist Consultation: A Review of the Literature

**DOI:** 10.4021/gr350w

**Published:** 2011-09-20

**Authors:** Carolyn De Coster, Monica Cepoiu-Martin, Carla Nash, Tom W Noseworthy

**Affiliations:** a Data Integration, Measurement & Reporting, Alberta Health Services, Canada; b Department of Community Health Sciences, Faculty of Medicine, University of Calgary, Calgary, Alberta, Canada; c Department of Internal Medicine, Faculty of Medicine, University of Calgary, Calgary, Alberta, Canada

**Keywords:** Family physicians, Gastroenterology, Gastrointestinal diseases, Referral and consultation, Review

## Abstract

**Background:**

Demands on gastroenterology are growing, as a result of the high prevalence of digestive diseases, the impact of colon cancer screening programs and an aging population. Prioritizing referrals to gastroenterology would assist in managing wait times. Our objectives were (1) to assess whether there were consistent criteria to guide referrals from family physicians for gastroenterological outpatient consultation and (2) to determine if there were different levels of urgency or priority in referral criteria.

**Methods:**

We conducted a scoping review, searching Medline, Embase and Cochrane databases from 1997 to 2009, using the terms referral, triage, consultation and at least one from a list of gastroenterology-specific search terms. Of 2978 initial results, 51 papers were retrieved, and 20 were retained after review by two reviewers. Additional publications were identified through hand searches of retained papers, website searches and nomination by a panel of specialists.

**Results:**

Thirty-four papers, reports or websites were retained. No referral criteria covered the spectrum of disorders that might be referred by family physicians to gastroenterologists. Criteria for referral were most commonly listed for suspected colorectal cancer, followed by suspected upper GI cancer, hepatitis, and functional disorders.

**Conclusions:**

A clinical panel comprised of gastroenterologists and primary care providers, informed by this literature review, are completing the work of formulating a Gastroenterology Priority Referral Score, and plan to test the reliability and validity of the tool for determining the relative urgency for referral from primary care to gastroenterology.

## Introduction

Demands on gastroenterology are growing. Digestive cancers are the second leading cause of cancer deaths in Canada. Furthermore, Canada has the highest incidence of both gastrointestinal ulcers and inflammatory bowel disease in the world and the prevalence of medically diagnosed bowel disorders has doubled in the past decade [1]. International data reveal that Canada and the UK rank fourth and fifth, respectively, among five countries in terms of gastroenterologists per 100,000 population. It is projected that the number of gastroenterologists will fall by 15% in the next 10 years unless training positions are increased [2].

A 2005 audit of Canadian gastroenterologists revealed that median wait times from primary care referral to investigation were longer than consensus targets set by the Canadian Association of Gastroenterology for seven digestive diseases [3]. Of note, two conditions had two-week targets: high likelihood of cancer based on imaging or physical exam and significant active inflammatory bowel disease (IBD). The cancer median waiting time was relatively close to the target: 26 days (interquartile range (IQR) 8-56 days), but the median wait for active IBD far exceeded the target: 101 days (IQR 35-209 days).

It is important to remember that gastroenterologists deal with a number of conditions affecting the luminal digestive tract (oesophagus, stomach, intestines) as well as biliary tract disease, pancreas disorders, liver disease and functional disorders. Given the scope of gastroenterologic outpatient consultation and long waiting times, we scoped the literature to assess whether there were consistent criteria to guide referrals from primary care to specialist consultation usually provided by a gastroenterologist. Furthermore we explored whether there were different levels of urgency or priority in referral criteria. This paper describes the findings of the review.

## Materials and Methods

Scoping reviews systematically synthesize a wide range of research and non-research material, identifying key concepts, sources of evidence and gaps in the research [4-5]. Steps in a scoping review are: identifying the research question, identifying relevant studies, study selection, charting the data, collating and summarizing the results and consultation [6-7].

**Figure 1:**
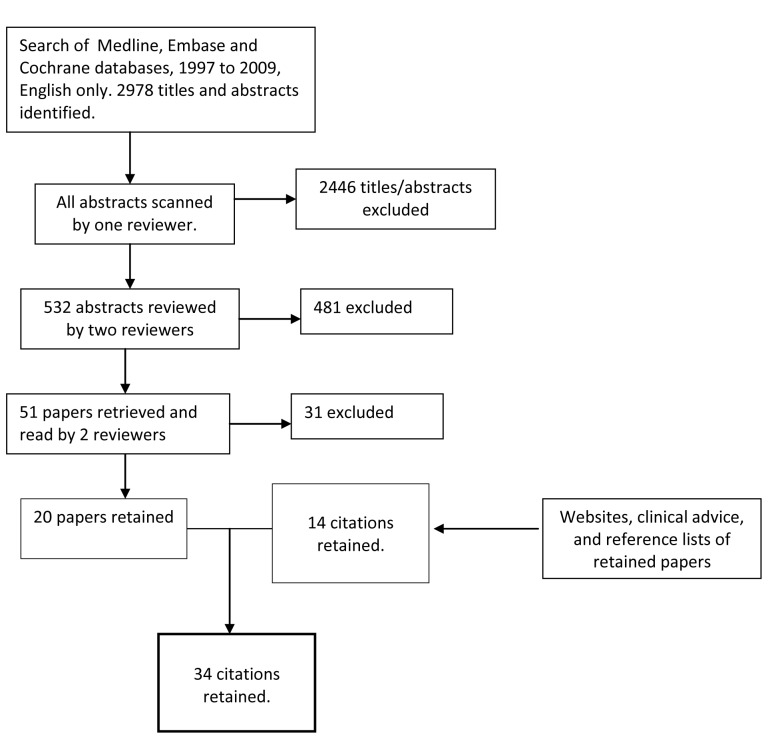
Diagram of literature search strategy for gastroenterology referral tool.

An academic research librarian searched Medline, Embase and Cochrane databases from 1997 to 2009, English only, using the terms referral, triage, consultation AND at least one from a list of gastroenterology-specific search terms (see appendix, web only file). The list was reviewed by a gastroenterologist and included disease terms such as cholelithiasis, oesophageal cancer, liver cirrhosis, rectal cancer, ulcerative colitis, as well as a list of symptoms such as rectal bleeding or jaundice. The search yielded 2978 abstracts and titles (Fig. 1).

**Figure 2:**
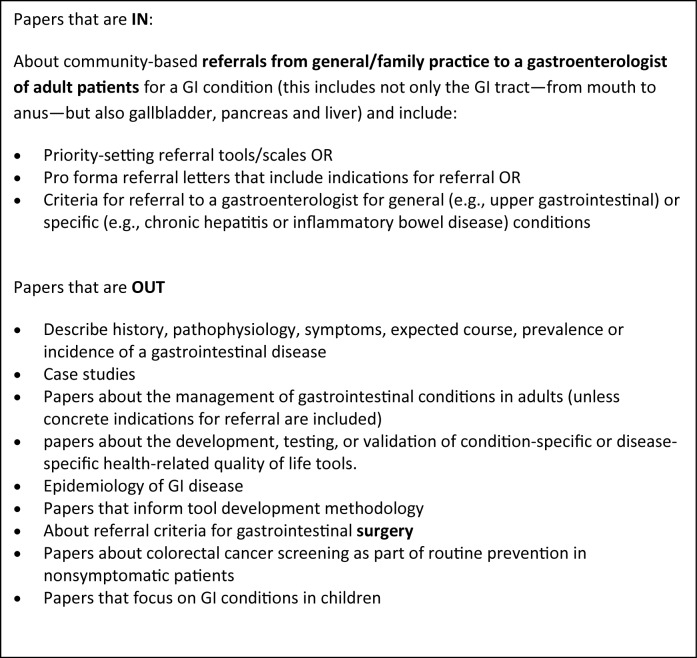
Inclusion and exclusion criteria.

Criteria for the selection of relevant abstracts were developed, tested and revised in an iterative process. The inclusion and exclusion criteria are described in Fig. 2. In scoping reviews, breadth is important but practicalities, such as time, budget and personnel resources, are important as well [7]. We knew from previous experience that many of the papers would not be relevant, and because of time constraints, each abstract was screened initially by one reviewer to determine if it should go on to a second in-depth review, with the understanding among team members to be as broadly inclusive as possible; 2446 abstracts were excluded at this stage. All remaining abstracts/titles were reviewed by at least two reviewers and rated as Yes, No or Possible. Abstracts or titles rated by at least one reviewer as Yes or Possible were reviewed by a third person. At the end of this process 51 papers had been identified for retrieval. These 51 papers were read in full by two reviewers; conflicts were read by a third reviewer and then discussed by all three readers. Twenty papers remained after this step.

References were hand-searched to identify any further citations. As well websites were consulted to look for referral prioritization guidelines or tools, including gastroenterology societies of Canada, Europe, Australia, United Kingdom and United States. This step yielded 12 additional citations. One reviewer abstracted the relevant data from each retained reference and compiled the results. In the consultation step, the resulting report was reviewed by five practising gastroenterologists who nominated two additional papers for inclusion. Thus 34 references were included in this systematic review.

## Results

Despite the high number of initial abstracts identified in our search, relatively few papers were retained. Table 1 identifies the 34 retained papers, reports or websites [8-41]. Criteria for referral were most commonly listed for suspected colorectal cancer[8, 10, 12, 13, 15-20, 22, 23, 25, 27, 28, 30, 31, 33, 35, 36, 38, 40]. Other GI conditions for which some referral criteria were found included suspected upper GI cancer[9, 24, 25, 27-30, 38, 39], hepatitis[11, 14, 26, 30], and functional disorders such as irritable bowel syndrome, dyspepsia, diarrhoea and constipation[21, 30, 32, 34, 37]. We found four papers on the management of a variety of important GI conditions, but they did not contain criteria for referral: inflammatory bowel disease[42], pancreatitis [43-44] and acute gastrointestinal blood loss[45]. The latter two require urgent hospitalization and would not go into the queue for outpatient gastroenterology consultation.

**Table 1:** List of Retained References with Referral Criteria for Colorectal Cancer, Upper Gastrointestinal Cancer, Hepatitis and Functional Bowel Disease

First Author	Country	Year	Citation	Colorec-tal Ca	Upper GI Ca	Hepatitis	Func-tional
Assoc of Coloproctology of Great Britian & Ireland[8]	UK	2007	http://www.acpgbi.org.uk/assets/documents/COLO_guides.pdf	•			
Allum WH[9]	UK	2002	Gut. 50 Suppl:v1-23		•		
Baig MK[10]	UK	2000	Hosp Med. 6197):452-3	•			
Barry J[11]	Ireland	2004	Ir J Med Sci. 173(3):145-9			•	
Barwick TW[12]	UK	2004	Colorectal Dis. 6(2):85-91	•			
Davies RJ[13]	UK	2002	Colorectal Dis. 4(1):28-30	•			
Fallon HJ[14]	US	1998	Consultant. 38(3):499			•	
Fijten GH[15]	Netherlands	1995	Fam Prac. 12(3):279-286	•			
Hamilton W[16]	UK	2004	Fam Pract. 21(1):990106	•			
Harinath G[17]	UK	2002	Colorectal Dis. 4(2):115-7	•			
Hodder RJ18]	UK	2005	Ann R Coll Surg Engl. 87(6):419-26	•			
Jiwa M[19]	Australia	2009	Quality in Primary Care. 17:31-36	•			
John SK[20]	UK	2008	Br J Surg. 95(4):506-14	•			
Jones J[21]	UK	2004	Gut. 47(Suppl 2): 1-19				•
Lawrenson R[22]	UK	2006	Eur J Cancer Care. (Engl) 15(3): 267-71	•			
Longstreth GF[41]	Multiple	2006	Gastroenterology 130:1480-91				•
Majumdar SR[23]	US	1999	Am J Gastroenterol. 94(10):3039-45	•			
Melleney EM[24]	UK	2004	Dysphagia. 19(2):78-82		•		
Moayyedi P[25]	Canada	2006	www.fhs.mcmaster.ca/idrp/cihr_final_report.pdf	•	•		
Mostert MC[26]	Netherlands	2004	J Hepatol. 41(6): 1026-1030			•	
Newton JL[27]	UK	2001	Baillieres Best Pract Res Clin Gastroenterol. 15(6):1013-25	•	•		
National Health Service[28]	UK	2000	http://www.dh.gov.uk/en/Publicationsandstatistics/Publications/PublicationsPolicyAndGuidance/DH_4008746	•	•		
Panter SJ[29]	UK	2004	Br J Gen Pract. 54(505):611-13		•		
Paterson WG[30]	Canada	2006	Can J Gastroenterol. 20(6):411-423	•	•	•	•
Selvachandran SN[31]	UK	2002	Lancet. 360(9329):278-83	•			
Spiller R[32]	UK	2007	Gut. 56(12):1770-1798				•
Tan YM[33]	Malaysia	2002	J Gastroenterol Hepatol. 17(3):281-4	•			
Thomas PD[34]	UK	2003	Gut. 52 Suppl 5:v1-15				•
Thompson MR[35]	UK	2003	BMJ. 327(7409):263-5	•			
Thompson MR[36]	UK	2007	Br J Surgery. 94:1260-1265.	•			
Tytgat G[37]	Multiple	1999	Eur J Gastroenterol Hepatol. 11(3): 223-30				•
UK Dept Health[38]	UK	2000	http://www.dh.gov.uk/en/Publicationsandstatistics/Publications/PublicationsPolicyAndGuidance/DH_4006963	•	•		
VanKerkhoven LA[39]	Netherlands	2007	Endoscopsy. 39(6):502-6.		•		
Wirral Hospital Trust[40]	UK	2001	Merseyside, UK: The Trust	•			

### Colorectal Cancer

In Canada, colorectal cancer is the third leading type of cancer [46]. Its primary symptoms – rectal bleeding, changes in bowel habit, abdominal pain, weight loss, unexplained anaemia – are common to many benign colorectal diseases, thus making it a challenge for family physicians to select patients who need referral for a gastroenterology consultation and for specialists to assess urgency [10]. Several efforts have therefore been made to identify alarm symptoms[8, 13, 15, 35, 38, 40], which typically include rectal bleeding with a change in bowel habit to looser or more frequent stools, older age and rectal bleeding without anal symptoms (i.e., soreness, discomfort, itching, lumps, prolapse, and pain), older age and change in bowel habit to looser or more frequent stools, palpable right-sided abdominal or rectal mass, and iron deficiency anaemia [38]. While there appears to be agreement that older age is an important consideration in assessing the probability of colorectal cancer, there is some disagreement about what age is relevant, with reported ranges from 45 to 65 years[13, 15, 38, 40].

In a systematic literature review, Moayyedi et al. [25] found no data on the overall accuracy of alarm features in detecting colorectal cancer. Pooled sensitivity, specificity and likelihood ratios and their 95% confidence intervals were estimated from the relevant papers for symptoms of rectal bleeding, change in bowel habit, diarrhoea, constipation, weight loss, anaemia and abdominal pain. Pooled positive likelihood ratios were highest for weight loss (2.42) and unexplained anaemia (2.36), followed by rectal bleeding (1.66), change in bowel habit (1.28). Positive likelihood ratios were low for diarrhoea (0.93), constipation (0.69) and abdominal pain (0.69).

Several authors suggested that combinations of two or more symptoms increases the level of urgency [12, 17, 22, 33, 36] Lawrenson et al. [22] pointed out that the absolute risk of colorectal cancer for patients presenting with a combination of two symptoms (anaemia and rectal bleeding, anaemia and changes in bowel habit, or rectal bleeding and changes in bowel habit) is about twice as high as those with any single symptom. In a prospective study of 8529 patients referred to a surgical clinic over a 12-year period, Thompson et al. [36] evaluated change in bowel habit, rectal bleeding, abdominal pain and perianal symptoms along with age as predictors of colorectal cancer. The highest positive predictive values were found with rectal bleeding and change in bowel habit, especially in the absence of perianal symptoms (19.7%). Perianal symptoms were protective, while abdominal pain was neither predictive nor protective. These findings were also correlated with age, suggesting that age in combination with the presence or absence of these three symptoms could guide practitioners in determining who should be referred more urgently. John et al. [20] validated an electronic referral protocol (e-RP) which assigned an urgency level depending on patient age, medical history and cluster of symptoms. The e-RP seemed to perform better than physician assessment at assigning urgency level and had the potential to reduce the rate of emergency presentation of colorectal cancer from 16% to 9%.

The use of high-risk symptoms and signs as criteria for urgent referrals has led to overwhelming referral rates in the UK. In 2002, Selvachandran et al. [31] evaluated the sensitivity and specificity of the Patient Consultation Questionnaire. This questionnaire was designed to obtain a comprehensive clinical history and included the following factors: age and sex, blood per rectum, change in bowel habit, tenesmus, urgency, and incomplete emptying, perianal symptoms, abdominal symptoms, weight loss, loss of appetite, tiredness, family history and relevant medical history. This questionnaire produces a Weighted Numerical Score (WNS). The researchers determined that a WNS over 60 is the best criteria to prioritize patients with colorectal symptoms. At this cut-off point, the population urgently referred includes not only patients with cancers but also patients with benign diseases that need urgent management, such as ulcerative colitis and polyps. The use of this questionnaire has been supported by others[18, 47]. An evaluation of the WNS compared with several other tools [13, 15, 23, 35, 40] concluded that the WNS with a threshold of 50 had a comparable sensitivity but a higher specificity than the rest of the tools in detecting cancer, yielding the lowest referral rate. Moreover, the WNS enabled the prioritization of other relatively severe colorectal diseases.

### Upper Gastrointestinal Cancer

The UK Department of Health’s [9, 38] two-week referral rule for suspected upper GI cancer includes: dysphagia, jaundice, upper abdominal pain, dyspepsia combined with alarm symptoms (weight loss, anaemia, vomiting), high-risk features in age 55+ (onset less than one year ago, or continuous symptoms since onset), or risk factors (family history of upper GI cancer in more than two first degree relatives, Barrett’s oesophagus, pernicious anaemia, peptic ulcer surgery more than 20 years ago, known dysplasia, atrophic gastritis or intestinal metaplasia).

These criteria lack both sensitivity and specificity: they would identify only 72% of cancer patients at their first visit to a GP, but result in many urgent referrals for upper GI endoscopy, most of whom will be found to have benign disease [29]. One chart audit on 396 patients referred with dysphagia found that 15% (60/396) of them did not have dysphagia, and 10% (41/396) were found to have cancer. Negative predictors of cancer were the presence of heartburn and having dysphagia for more than one year [24]. Another study concluded that alarm symptoms, defined as weight loss, dysphagia or melena/haematemesis, had a 4% positive predictive value for finding cancer, but that many patients with cancer identified using these guidelines are untreatable [39].

### General Non-Cancer Referrals

Beyond cancer, there are many reasons for referral to gastroenterology from primary care, with varying degrees of urgency for referral. Much of the literature focuses on specific diseases. The Guidelines for referral of patients with chronic Hepatitis B (HBV) were developed and evaluated in Rotterdam. The Dutch standard was for family physicians to refer all patients with chronic HBV to a specialist [26]. However the evaluation demonstrated that the guidelines enabled family physicians to select only those patients with chronic active HBV for specialist referral, thus avoiding referrals for chronic inactive infection since no treatment was indicated for these patients. This is in contrast to the Canadian consensus which recommends that patients with chronic viral hepatitis should be seen within two months, largely because of patient anxiety [30].

In 1998, Fallon recommended that all patients newly diagnosed with hepatitis C should be referred to a specialist, except for those with undetectable HCV RNA levels, an indication of spontaneous recovery [14]. More recently, the Dublin Area Hepatitis C Initiative Group created criteria to help predict which patients with hepatitis C from drug use would benefit from therapy [11]. These criteria include HCV and PCR/phenotype testing, clinical and laboratory evaluation of liver status, freedom from unstable drug use, psychiatric history and social stability.

Inflammatory bowel disease (IBD) includes ulcerative colitis and Crohn’s disease. The most common symptom of ulcerative colitis is bloody diarrhoea; less common are colicky abdominal pain, urgency and tenesmus [42]. Crohn’s disease symptoms include abdominal pain, diarrhoea, weight loss, malaise, anorexia and fever. Although we found guidelines for the management of patients with IBD, there were no criteria for referral. A vexing problem is distinguishing between IBD with its morbidity and need for optimal therapy, from functional bowel disorders.

Functional bowel disorders, such as irritable bowel syndrome, dyspepsia and chronic constipation, are the most common medical conditions seen in both primary care and gastroenterology [48]. The prevalence of IBS ranges from 2.5% to 20%, depending on the criteria set used [32]. The Rome III diagnostic criteria are the most up-to-date: recurrent abdominal pain or discomfort at least 3 days a month in the past 3 months, associated with two or more of the following: improvement with defecation, onset associated with a change in frequency of stool, or onset associated with a change in appearance of stool [41]. Referral for IBS should occur if symptoms are atypical or alarming (such as age 50 or older, short history of symptoms, documented weight loss, nocturnal symptoms, male sex, family history of colon cancer, anaemia, rectal bleeding, recent antibiotic use), if there is uncertainty about the diagnosis, or if patient concerns have not been successfully allayed in the primary care physician visit [21, 32].

In patients with dyspepsia, Tytgat et al. [37] summarized risk factors that should prompt the referral of these patients for early endoscopy: the presence of alarm symptoms (anaemia or evidence of bleeding, severe or persistent pain, painful swallowing, difficulty swallowing, recurrent or persistent vomiting, anorexia, weight loss); presentation with first-time dyspepsia or altered symptoms over the age of 55; previous history of peptic ulcer disease; current ulcer disease and recent evidence of substantial gastrointestinal bleeding; use of NSAIDs; and other risk factors (heavy smoking, alcohol abuse). Reflux-like symptoms (regurgitation, reflux or heartburn) have been shown to have a 33% positive predictive value for finding reflux oesophagitis during open-access endoscopy [39].

## Discussion

Our review of the literature did not elucidate any tools or guidelines that would prioritize the broad scope of patients that may be referred by primary care physicians to gastroenterologists. There appears to be some consensus about alarm symptoms that should prompt an urgent referral. For lower GI conditions, these include rectal bleeding, anaemia and change in bowel habit, especially if at least two of these symptoms are present at the same time. For upper GI disease, they are progressive dysphagia, haematemesis, or dyspepsia with weight loss or anaemia or vomiting. Using these criteria to designate patients as urgent could result in overwhelming gastroenterological services; even though most patients thus referred will have benign disease, it is important that patients have reasonable access for specialist evaluation when referral is indicated. Managing the queue is a challenge for many gastroenterology practices.

In Canada, colorectal cancer is the second leading cause of cancer deaths in males and third in females [46]. Earlier detection through screening programs or referral guidelines thus has the potential to save many lives. When the UK implemented its two-week-wait rule for consultations for patients with alarm symptoms, many GI practices were overwhelmed. The literature demonstrates that combinations of symptoms increase the likelihood of cancer detection, suggesting that combinations of symptoms, together with age, should be incorporated into referral guidelines and may help in assessing urgency.

In 2006, a Consensus Group of Canadian gastroenterologists and hepatologists published medically acceptable wait times for access to specialist gastroenterological care [30]. Four urgency categories were defined: within 24 hours, within 2 weeks, within 2 months and within 6 months. Each category lists from 4 to 10 symptoms or conditions. This work goes some distance towards identifying urgency criteria spanning the scope of referrals. However, its four urgency bands are relatively broad. Furthermore, each wait time target can be met by the presence of only one condition or symptom, and does not allow for the additional urgency that may be implied by the presence of a constellation of symptoms.

Development of a prioritization referral tool would benefit both primary care providers and gastroenterologists. For primary care providers, the tool would help to standardize the referral process, reducing the frustration of multiple forms and referral requirements. It should be easy to use, and if tests are to be performed, they should not be expensive, complicated, or restricted to specialist use [49]. For gastroenterologists, required information will be available; studies show that 50% or more of referral letters are missing information that specialists require [50-54]. Referrals would be prioritized in order of urgency in a transparent process. To this end, a clinical panel comprised of gastroenterologists and primary care providers, informed by this literature review, are completing the work of formulating a Gastroenterology Priority Referral Score, and plan to test the reliability and validity of the tool for determining the relative urgency for referral from primary care to gastroenterology.
